# Effectiveness of Global Optimisation and Direct Kinematics in Predicting Surgical Outcome in Children with Cerebral Palsy

**DOI:** 10.3390/life11121306

**Published:** 2021-11-27

**Authors:** Claude Fiifi Hayford, Emma Pratt, John P. Cashman, Owain G. Evans, Claudia Mazzà

**Affiliations:** 1 Department of Mechanical Engineering, INSIGNEO Institute for in Silico Medicine, University of Sheffield, Sheffield S10 2TN, UK; c.mazza@sheffield.ac.uk; 2 Gait Analysis Laboratory, Sheffield Children’s Hospital, Sheffield S10 5DP, UK; emmapratt@nhs.net (E.P.); j.cashman@nhs.net (J.P.C.); owain.evans3@nhs.net (O.G.E.)

**Keywords:** gait analysis, direct kinematics, global optimisation, gait profile score, cerebral palsy

## Abstract

Multibody optimisation approaches have not seen much use in routine clinical applications despite evidence of improvements in modelling through a reduction in soft tissue artifacts compared to the standard gait analysis technique of direct kinematics. To inform clinical use, this study investigated the consistency with which both approaches predicted post-surgical outcomes, using changes in Gait Profile Score (GPS) when compared to a clinical assessment of outcome that did not include the 3D gait data. Retrospective three-dimensional motion capture data were utilised from 34 typically developing children and 26 children with cerebral palsy who underwent femoral derotation osteotomies as part of Single Event Multi-Level Surgeries. Results indicated that while, as expected, the GPS estimated from the two methods were numerically different, they were strongly correlated (Spearman’s ρ = 0.93), and no significant differences were observed between their estimations of change in GPS after surgery. The two scores equivalently classified a worsening or improvement in the gait quality in 93% of the cases. When compared with the clinical classification of responders versus non-responders to the intervention, an equivalent performance was found for the two approaches, with 27/41 and 28/41 cases in agreement with the clinical judgement for multibody optimisation and direct kinematics, respectively. With this equivalent performance to the direct kinematics approach and the benefit of being less sensitive to skin artefact and allowing additional analysis such as estimation of musculotendon lengths and joint contact forces, multibody optimisation has the potential to improve the clinical decision-making process in children with cerebral palsy.

## 1. Introduction

Three-dimensional clinical gait analysis (CGA) use is widespread in the diagnosis and treatment of movement abnormalities particularly in people with cerebral palsy, where it forms a part of the clinical decision-making process [1,2]. When considering surgical interventions, studies report up to 92% agreement between pre-operative CGA recommendations and the actual surgery performed [3,4], underlying its importance in the clinical setting. CGA produces a variety of kinematic and kinetic output variables, primarily including estimates of joint angles, moments, and powers during the gait cycle of a patient.

The most common approach to estimating joint kinematics in CGA is the so-called conventional gait model, which is based on the Davis protocol [5] and also implemented in the Plug-in-Gait model (PiG, Vicon, Oxford, UK). In this approach, experimental markers are attached to the body and used to determine the orientation of the anatomical segments and the 3D intersegmental joint kinematics. In the so-called direct kinematics approach, the latter are calculated using Cardan angles, i.e., a sequence of rotations about three different and mutually perpendicular axes, that rotate a distal segment with respect to a proximal segment [5,6,7]. While this approach enjoys widespread clinical acceptance and use, it has been shown to have limitations, especially in regard to error due to soft tissue artefacts [8]. Additionally, when using this approach, bony segment dimensions might vary as a result of being defined by the time variant distances between joint centres [8] arising from the limited marker set of the PiG, which uses the same markers to define adjacent segment lengths. This latter issue precludes the subsequent use of the same model when estimating other relevant metrics, such as the muscle length and the internal forces obtainable through musculoskeletal modelling techniques [8]. Multibody optimisation, also referred to as global optimisation, has hence been proposed as an alternative approach for estimating joint kinematics. This method simultaneously determines the orientation of a constrained skeletal model while minimising measurement errors in marker locations. It addresses some of the challenges of the PiG model, in particular minimising skin tissue artefacts [9], and has been shown to provide reliable estimates of gait kinematics [10,11]. However, its successful adoption in routine assessment of pathological gait is yet to be determined due to limited implementation in commercial software packages and limitations associated to the simplifications made within the reference joint models, such as the use of minimal degrees of freedom as well as complexity and time constraints [12,13]. A comparison between estimates obtained using the direct and global optimisation approaches described above as applied to gait data from both typically developing children and children with cerebral palsy showed that the choice of approach resulted in root mean square differences between the two kinematic outputs of less than 1° when keeping the same anatomical model (i.e., the same degrees of freedoms for the joint models and segments anatomical definitions) [14]. The same study showed that among all factors, 94% of the variations are to be attributed to the anatomical model.

Kinematic outputs from CGA cover multiple joints, anatomical planes, and span across the whole gait cycle. This multiplicity of interdependent information can benefit from being summarised into a single measure to aid interpretation [15]. One such measure is the Gait Profile Score proposed by Baker et al. [16] which provides a summary index of overall gait quality or pathology and has been shown to have high clinical validity in terms of its relationship with other clinical measures and the ability to quantify changes in gait patterns and the effects of treatment interventions in pathological populations [17,18,19,20]. The formulation of the GPS also allows for identifying the aspect of the kinematics contributing to the deviation from normal [16]. While use of the GPS is well documented with the PiG model, to the best of our knowledge, no studies have calculated this metric using outputs from other modelling approaches, including global optimisation. Furthermore, independently of the modelling approach, the GPS has not been previously evaluated with respect to its agreement with non-3D data-based clinical judgement of surgical outcome in a pathological population. Hence, the aim of this paper is to evaluate the agreement between GPS and clinical data-based judgment in estimating surgical post-intervention outcomes when the GPS is calculated from kinematic outcomes obtained using either the global optimisation or the direct kinematic approach. In particular, as a paradigmatic case where using global optimisation could be particularly challenging, we focused on investigating the outcome of femoral derotation osteotomy, which is a surgical procedure aiming at correcting excessive femoral anteversion.

## 2. Methods

### 2.1. Data Description

This study retrospectively analysed the anonymised data of 26 children with CP (age: 8.8 ± 3.0 years, mass: 26.1 ± 9.2 kg, height: 1.26 ± 0.16 m at pre-intervention observation) from the Sheffield Children’s Hospital Gait Laboratory database. Ethics was approved by the South Central—Oxford B Research Ethics Committee (19/SC/0329).

Participants were selected based on the following criteria:

1. Diagnosis of cerebral palsy;
2. Gross Motor Function Classification System (GMFCS) I–III;
3. Femoral Derotation Osteotomy as part of Single Event Multi-Level Surgery (FDO-SEMLS);
4. Availability of at least one pre- and post-intervention 3D gait analysis capture.

The CP cohort had gait analysis on average 13.1 ± 9.1 months prior to FDO-SEMLS and again 31.3 ± 18.5 months after the surgery. Additionally, retrospective control data of 34 typically developing (TD) participants (age: 15.9 ± 9.8 years, mass: 47.5 ± 20.7 kg, height: 1.53 ± 0.22 m) were analysed. Participants in the TD cohort were divided into three subgroups: children (<10 years), teenagers (<16 years), and young adults (≥16 years) as detailed in Table 1. For all groups, CGA was performed using a Vicon system (Vicon, Oxford, UK) and comprised one static and at least three walking trials. Markers were placed on the lower limbs using the Plug-In Gait marker set. A Knee Alignment Device (KAD) was utilised in the standing trial defining the knee flexion axis and a thigh rotation offset angle relative to the applied thigh wand. The KAD was subsequently replaced by a single lateral knee marker (KNE) in the walking trials.

### 2.2. Calculation of the Joint Kinematics

**Figure 1:**
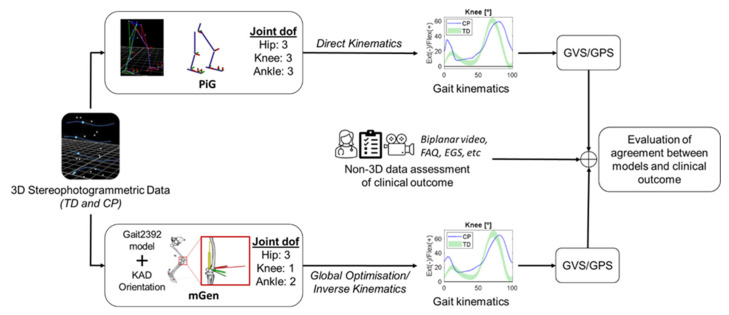
Flowchart showing workflow of analysis.

To achieve the study objective, two workflows (Figure 1) were followed. Firstly, the gait2392 lower-limb model bundled with Opensim3.3 [21] was adapted to each participant’s characteristics. This model has three degrees of freedom at the hip, one at the knee and hinges for both the tibiotalar joint and subtalar joint. The metatarsophalangeal joint was locked. Prior to scaling the model to each participant’s anthropometry, the orientation of the knee flexion axis in the transverse plane was modified with custom MATLAB (R2018b, Mathworks, MA, USA) scripts to match the alignment of a KAD placed on the participant during a static trial. The applied rotation to the gait2392 model’s knee flexion axis was compared between TD, CP pre-surgery, and CP post-surgery with a Kruskal–Wallis test followed by a post hoc Dunn’s multiple comparison test. Then, this modified model was scaled following recommended procedures [22]. Briefly, scale factors were calculated from surface marker positions and calculated joint centres. The pelvis was scaled anisotropically, whereas the femur and tibia were scaled linearly using distances between the hip and knee joint centres and knee and ankle joint centre, respectively. The modified scaled generic models (mGen) were subsequently used to simulate the participants’ gait using the Inverse Kinematics tool in OpenSim3.3 to estimate their joint kinematics over the gait cycle. In addition, the foot progression angle (FPA) over the gait cycle was calculated. To this purpose, the OpenSim “Analyze” tool was used to estimate the model body kinematics, from which the direction of progression was defined as the vector from the pelvis centre of mass position at initial heel strike to its position at end of the gait cycle. The foot vector was defined as the line joining the ankle joint centre to the model TOE marker [23]. The FPA was calculated as the angle between the direction of progression and the foot axis projected in the transverse plane using custom MATLAB scripts.

The second workflow followed the standard clinical protocol of the Sheffield Children’s Hospital Gait Laboratory for PiG model data analysis using Nexus (Vicon, Oxford, UK). Post processing with thigh rotation offset adjustment [24] was completed using laboratory standard protocols to optimise the knee axis alignment where necessary.

### 2.3. Data Analysis

From the output of both workflows, eight joint angles (pelvic tilt, pelvic list/obliquity, pelvic rotation, hip flexion, hip abduction, hip rotation, knee flexion, and ankle dorsiflexion) and the FPA were extracted, and time was normalised to 100% of the gait cycle using custom scripts in MATLAB. Additionally, for each subject, the mean kinematic waveform of the three gait trials for each limb was calculated.

The kinematics estimated with the two workflows were statistically compared using the non-parametric paired sample t-test option of the 1-dimensional statistical parametric package [25] in MATLAB. A Bonferroni correction was applied for multiple comparisons, and α was set to 0.0056 (9 pairwise comparisons). The root mean square difference (RMSD) was calculated between individual trials’ kinematic waveform from PiG and mGen for each subject and averaged to quantify the effect of the chosen approach on the joint angle estimates. Similarly, the coefficient of determination (R^2^) was calculated for individual trials with the linear fit method between PiG and mGen and averaged per subject for each of the nine kinematic variables as a measure of curve similarity.

The GPS was also calculated for all subjects using the data from the age-matched controls and the mean kinematic waveform outputs from both mGen and PiG. The GPS is calculated separately for each limb and is based on the Gait Variable Score (GVS), which is the root mean square (RMS) difference between the gait vector of a patient and the mean gait vector of a non-pathological group. These gait vectors include nine key kinematic variables: pelvic tilt, pelvic list/obliquity, pelvic rotation, hip flexion, hip abduction, hip rotation, knee flexion, ankle dorsiflexion, and foot progression angle. Formally, the GVS and GPS were calculated as described in Equations (1) and (2), respectively.

(1) $$GVS_{i}=\sqrt{\frac{1}{T}\;\overset{T}{\underset{t=1}{\sum }}{\left(x_{i,t}-x_{i,t}^{ref}\right)}^{2}}$$

where $GVS_{i}$ is the Gait Variable Score of the kinematic variable i, t is a specific instance in the gait cycle, T is the total number of points in the cycle, $x_{i,t}$ is the value of kinematic variable i at instance t, and $x_{i,t}^{ref}$ is the mean of that variable at the same time point for a reference population.

(2) $$GPS=\sqrt{\frac{1}{N}\overset{N}{\underset{i=1}{\sum }}GVS_{i}{}^{2}}\;$$

where N is the total number of kinematic variables used and i is the ith kinematic variable used.

Data were checked for normality, and appropriate statistical tests were used subsequently. Consistency in GPS estimated with mGen and PiG was assessed using Spearman’s correlation coefficient (ρ). A Wilcoxon rank test was performed to determine significant differences between the two groups of GPS estimates. Additionally, the Symmetry Index (SI) [26] was used to calculate left/right limb asymmetry in GPS values for both models and compared using the Pearson’s correlation coefficient. To test the agreement between models when estimating changes in GPS (Δ_t_GPS) after FDO-SEMLS, a Bland–Altman analysis was used. Δ_t_GPS were normally distributed, and a paired sample t-test was used to determine if model estimates of Δ_t_GPS were significantly different. To understand the contributing factors to any differences in Δ_t_GPS estimated by both models, the above-described analyses were applied also to the nine components of the GPS.

To determine a classification of improvement (responders) from 3D gait analysis data using both mGen and PiG estimates of GPS, change in GPS (Δ_t_GPS) from pre- to post-FDO-SEMLS for each model was calculated, after which a criterion of Δ_t_GPS ≤ −1.6 (minimal clinically important difference for GPS [27]) was applied. This 1.6° threshold reflects the mean difference between GPS values of children classified in adjacent levels of the Functional Assessment Questionnaire measure of functional mobility. Participants with Δ_t_GPS values not satisfying this criterion were classified as non-responders.

The process for clinically adjudging participants as responders or non-responders to FDO-SEMLS was based on consensus between two consultant orthopaedic surgeons and the team of gait laboratory staff (including clinical scientists and physiotherapists). This process involved a review of clinical examination findings and clinical dictations as well as video recordings looking at components of the Edinburgh Gait Score (EGS) [28], a validated visual analysis scale, that pertained to the desired correction from the FDO. In this regard, the video analysis emphasized hip rotation (using the knee progression angle in stance with consideration of transverse plane alignment as a surrogate) and the foot progression angle in stance. One clinical scientist completed the classifications, and these decisions were reviewed by the consultant orthopaedic surgeons. The measured kinematics and report from 3D gait analysis were not used in this process. Only the limbs that underwent the surgery were considered for each participant.

## 3. Results

**Figure 2:**
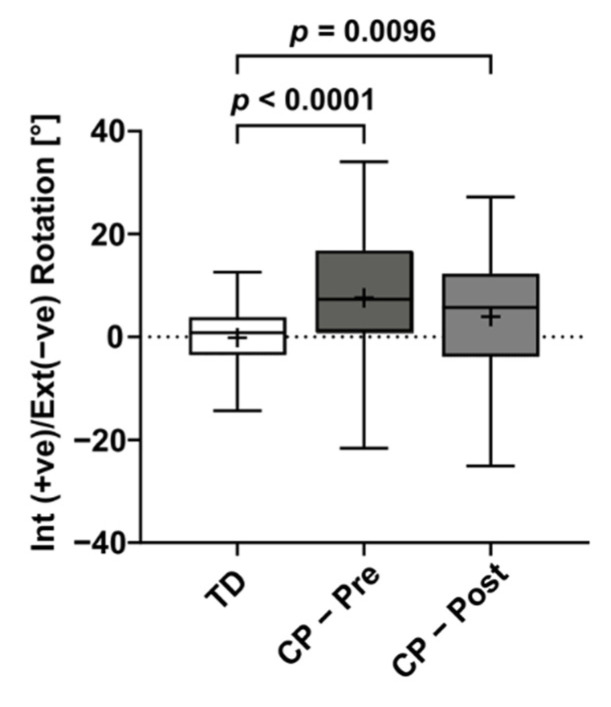
Box plot of knee flexion axis corrections estimated from the KAD and applied to mGen for the TD and CP participants. *p*-values indicate significant differences as highlighted by a post hoc Dunn’s multiple comparison test comparing TD, CP pre, and CP post data.

**Figure 3:**
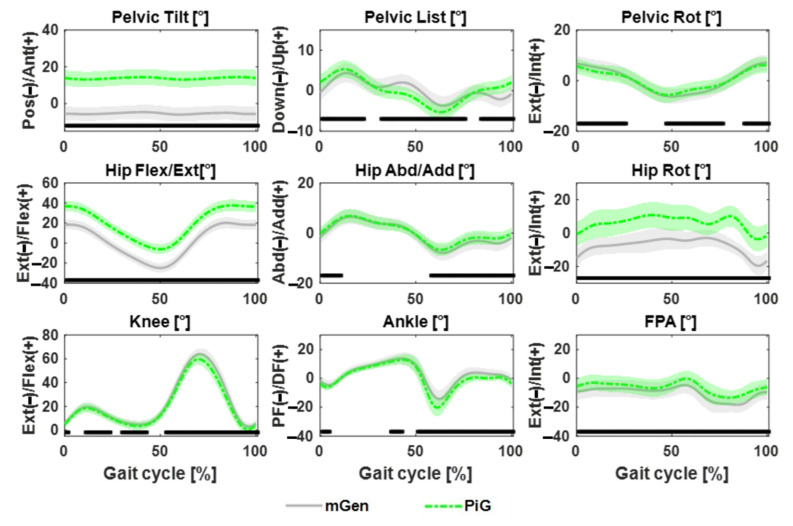
Mean joint kinematic waveforms for 68 limbs from 34 TD participants estimated with PiG and mGen models. Shaded bands indicate 1SD with significant difference shown by bottom black bars.

**Figure 4:**
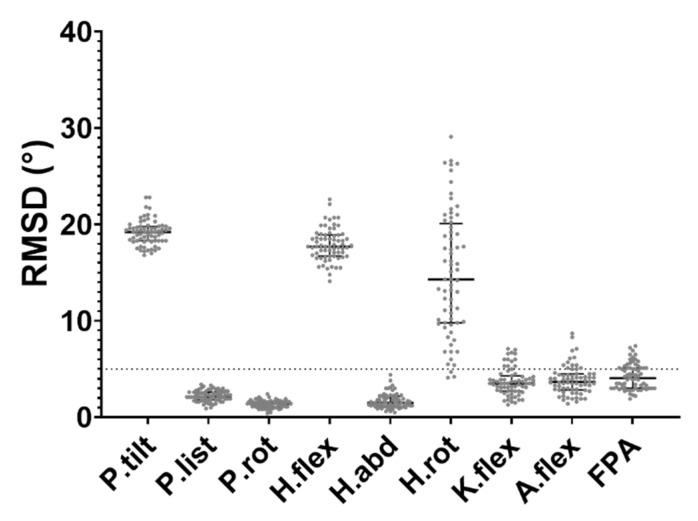
Distribution of RMSD of joint kinematics between PiG and mGen models for TD participants. Bars represent median and interquartile range. Dotted line at 5° represents clinically acceptable threshold of data error [29].

The KAD-based modifications to the knee flexion axis were found to be significantly different between TD and CP groups at both pre- and post-intervention (Figure 2). There was also large variation within the CP cohort in terms of the modification that was applied to the generic model, although this did not change after the surgery. Figure 3 shows the kinematic waveforms estimated by PiG and mGen for the TD participants. Mean inter-trial standard deviation for the kinematics waveforms are shown in Supplementary Figures S3 and S4 for CP and TD, respectively. Joint kinematics over the gait cycle estimated by the two models were highly correlated (minimum average R^2^: 0.83 ± 0.10) with the exception of pelvic list/obliquity and hip internal/external rotation (average R^2^: 0.61 ± 0.23 and 0.55 ± 0.18, respectively). Additionally, the joint kinematics for pelvic tilt, hip flexion, and hip rotation had large differences between model estimates (RMSD > 5°). These results were similar for the CP group (see Supplementary Data, Figure S1). The distribution of RMSD quantifying the differences between joint angles estimated by PiG and mGen for the TD participants is shown in Figure 4 (data for CP cohort shown in Supplementary Figure S2). The mean RMSD over all analysed joints was 7.7 ± 0.9° and 8.0 ± 1.3° for TD and CP, respectively. Hip rotation had the largest variability in RMSD for both TD and CP (IQR = 10.0° and 7.7°, respectively).

**Figure 5:**
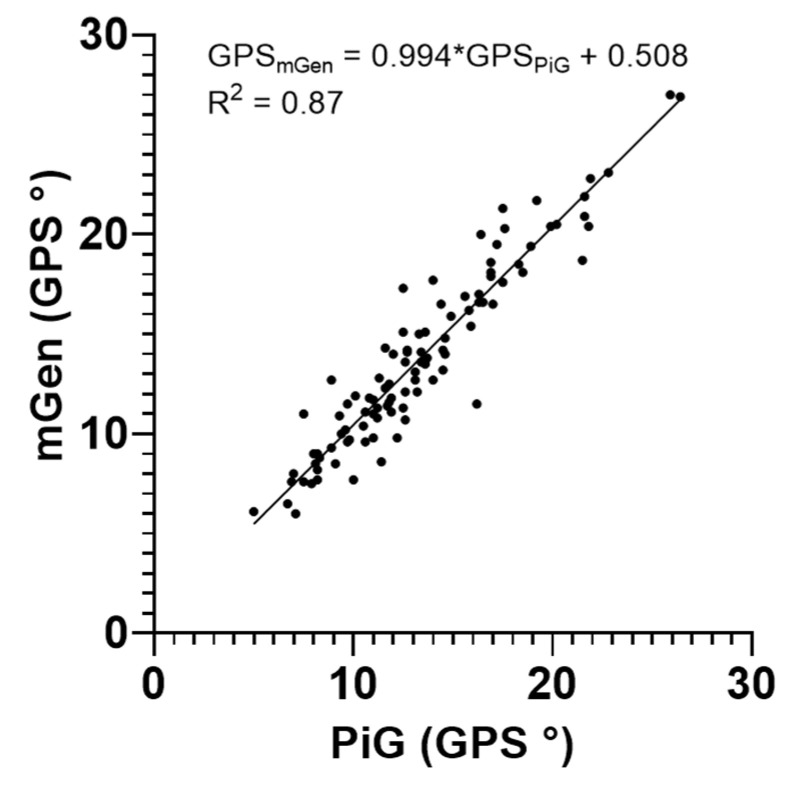
Correlation between the GPS estimated with PiG and mGen for all subject limbs and observations.

The comparison of the GPS calculated from the kinematic output of the two approaches indicated agreement in values with a mean absolute difference of 1.1° (range: 0.0° to 4.8°). Figure 5 shows the strength of the relationship between PiG and mGen with a Spearman ρ of 0.93 (*p*-value < 0.0001). However, the GPS values estimated by PiG and mGen were found to be significantly different (*p*-value = 0.002) using the Wilcoxon test. Both models reported on average a similar Symmetry Index (4.8 ± 23.6% and 3.2 ± 24.0% for PiG and mGen, respectively) with a Pearson correlation coefficient r of 0.90 between the two.

**Figure 6:**
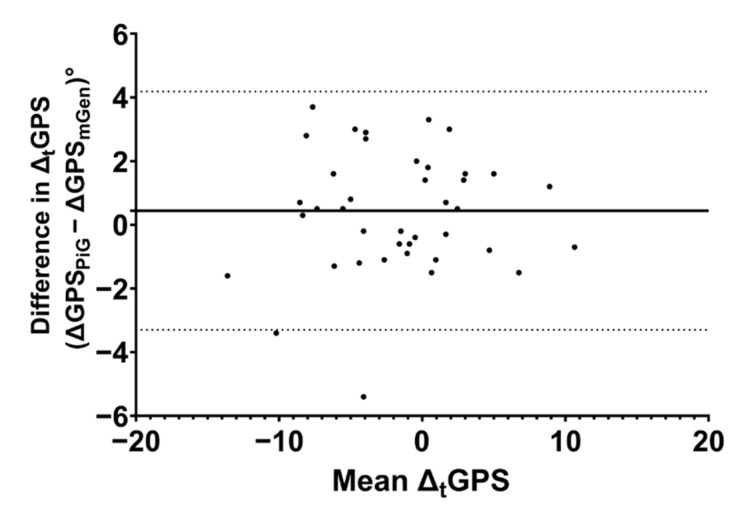
Bland–Altman plot of changes in GPS estimated by PiG and mGen. Solid and dotted lines represent the mean difference and limits of agreement, respectively.

Changes in gait kinematics pre- and post-intervention, as assessed by Δ_t_GPS, were not significantly different when calculated using the PiG or the mGen (*p*-value = 0.14, paired *t*-test, Supplementary Table S1), although PiG estimated generally higher Δ_t_GPS than mGen. The Bland–Altman analysis (Figure 6) showed a bias in estimating Δ_t_GPS of 0.44° (Limits of Agreement: −3.29° to 4.18°).

**Figure 7:**
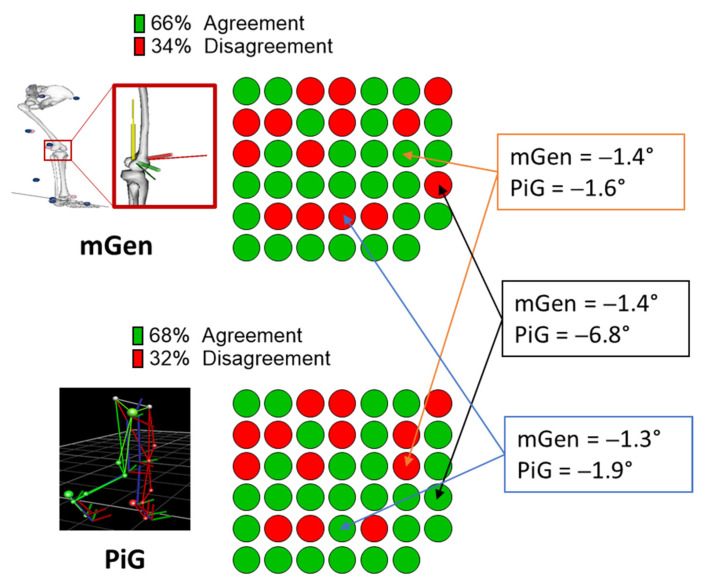
Agreement of clinical classification of CP participants after surgery with PiG and mGen-based classification from GPS values for 41 limbs analysed. The boxes represent the three cases where there was a difference (values within box) between model predictions of outcome with similar coloured arrows pointing to the prediction from mGen and PiG.

Results from comparing the clinical judgement with the models’ classifications are shown in Figure 7. Overall, there was >60% alignment of both PiG and mGen with the clinical judgement. When not considering what the clinical judgement was, both models predicted the same outcome in greater than 90% of cases (38 out of 41 limbs). For the three limbs where the prediction of classification differed between models, differences between model estimates of Δ_t_GPS were negligible with the exception of one case (0.2°, 0.6°, and 5.4°, respectively). Additionally, for these three cases, both models were consistent in their estimation of post FDO-SEMLS increase/decrease in GPS.

## 4. Discussion

This retrospective study sought to evaluate the agreement between non-3D data-based clinical judgement of surgical outcome and kinematic-based outcomes estimated using either global optimisation or direct kinematics in children with CP after Femoral Derotation Osteotomy as part of Single Event Multi-Level Surgery (FDO-SEMLS).

Results showed that joint kinematics estimated by the two modelling approaches were highly correlated, although there existed differences in the values. The main difference between the two approaches were offsets for pelvic tilt, hip flexion, and hip rotation. This was consistent with earlier reported studies in children with CP that attributed these offsets to the differences in both the anatomical and joint axes definitions [14]. In the PiG, the pelvis anatomical coordinate system is oriented such that the anteroposterior axis lies in a plane defined by the pelvis markers (sacrum and two anterior iliac spines), whereas the axis for the mGen is oriented parallel to the horizontal. This led to a RMSD difference of about 10–15°, which was shown to reduce by adjustment of the anatomical and joint reference frames [10,11]. Similarly, in this study, an RMSD of 18 ± 1.5° in pelvic tilt between the PiG and mGen approaches was found. However, such model differences are not expected to influence the estimation of GPS, as the model definitions were kept consistent within each approach and its reference data.

Compared to the other joints, a larger range of values was observed in the hip rotation’s RMSD. This is most likely to be attributed to the differences in degrees of freedom observed between the two models. In fact, Horsak et al. [11] showed in a group of obese children that using three instead of one degree of freedom at the knee joint in mGen reduced the RMSD between mGen and PiG estimates of hip rotation. From their results, it could also be observed that the R^2^ improved with a 3-DoF mGen knee. In agreement with their findings, our analysis of these data in the TD cohort showed a significant decrease in the magnitude and variability of hip rotation RMSD when a 3-DoF knee joint was used in the mGen (see Supplementary Figure S5). An additional explanation for the observed range of values could be associated to the inaccuracies in the tracking of the thigh marker, which is the main driver of hip rotation when using global optimisation. While still within the recommended limits, this marker had on average the highest tracking root mean square error (1.88 ± 0.77 cm and 1.74 ± 0.62 cm for right and left thigh markers, respectively, Supplementary Table S3).

From our analysis, mGen and PiG models calculated similar values for the GPS with a mean absolute difference of 1.1° observed between the two model estimates, which was less than the cut-off for clinical significance. Additionally, the estimation of left and right asymmetry in GPS by mGen was captured similarly to the PiG (r = 0.90, Table S2 in Supplementary Materials). In comparing agreement in predictions of outcome after the FDO-SEMLS, it was shown that in 65.9% (27/41 limbs) of cases, the mGen predicted the same status for the patient as the clinical judgement. A similar agreement (68.3%, 28/41 limbs) was found for the PiG. The observed level of agreement with the clinical judgement for both models is in line with the notion that clinical assessment and CGA are relatively independent sources of information and should be used in tandem [30]. The clinical judgement of post-intervention outcome used in this study was based on clinical measures such as clinical examination, subjective reports (e.g., Gillette Functional Assessment Questionnaire), and video footage of the gait with no dependence on 3D gait analysis output. Placing this in context, studies have shown that changes in surgical recommendations after including gait analysis data occurred in 52% of cases [31], and the GPS is just one of the outputs from gait analysis. Although both models’ agreement with the clinical judgement was less than 70%, the classification of participants as responders or non-responders by mGen and PiG using Δ_t_GPS aligned in 38 out of 41 limbs compared. This relatively high concordance (92.7%) is important for the purpose of this study because it shows that the mGen is capable of predicting similar changes in gait kinematics after FDO-SEMLS as the PiG, despite different values of GPS. Notably, for the three cases that were not aligned in terms of agreement in predicted outcome, both models predicted the same direction of change in GPS in all cases and negligible differences between models’ estimates in all but one case (0.2°, 0.6° and 5.4°, respectively). Exploring the latter, the PiG was correctly aligned with the clinical judgement, and there was a large inter-model difference (>10°) between mGen and PiG in the GVS of hip rotation and ankle plantarflexion. This difference was also reflected in the RMSD in those variables between PiG and mGen for that case. Taken together, these results suggest mGen performs similarly to PiG in estimating surgical outcomes when using the GPS.

When looking at the transverse plane components of the GPS, changes in FPA were more likely to agree (>70%) with the clinical judgement for both mGen and PiG, and this resonated with the fact that the FPA was one of the main factors used to classify a response as per the criterion for classification. Using a machine learning approach, Schwartz et al. [32] showed that femoral derotation osteotomies have a causal effect on FPA in CP, and this result from our analysis goes to further justify the applied criterion for clinical classification. Interestingly, changes in the hip rotation component of the GPS showed that the mGen performed better (58.5% vs. 48.8%) than the PiG in its agreement with the clinical judgement. For these two aspects of importance for FDO-SEMLS, the mGen was marginally better than the PiG when compared to the clinical judgement.

With mGen showing near equivalence to the PiG with respect to the GPS, it can be argued that the use of global optimisation might be preferred if the aim is to provide additional benefit to the clinical decision-making process. In fact, this approach, besides improving the estimate of the joint kinematics [9,33], allows for the estimation of muscle tendon lengths and forces and joint reaction forces, which are parameters not directly accessible to the direct kinematics approach. A number of studies have reported using the output of PiG to drive a musculoskeletal model to obtain these variables [13,34]; however, as reported by Kainz and Schwartz [13], it is important to ensure consistency between the mGen and PiG, as this influences the estimates of muscle-tendon lengths obtained in this way.

The knee axis modification that was applied using information from the KAD was found to be significantly different between the TD and CP cohort, which was expected. On the other hand, there were no significant differences between the pre- and post-modifications made for the CP cohort. This could be attributable to the heterogenous nature of the CP limbs, as there were limbs that saw improvement or deterioration and others that were not operated on. Further analysis, requiring additional data, is warranted to investigate how the applied modifications could be used as a surrogate measure to clinical measures, such as the degree of femoral anteversion and hip passive range of motion.

The limitations of the current study are acknowledged. Changes in femoral or tibial torsions that were present in the CP participants were not fully captured by the mGen models. Personalisation of musculoskeletal geometry is known to improve estimates of joint kinematics [35] due to the influence on joint centre estimates and axis definitions. Nonetheless, this study attempted to approximate some aspect of personalisation in the mGen model, adapting the model to use the knee axis orientation defined with the KAD. An alternative approach of deforming the generic model using clinical measures of femoral anteversion has been proposed [36,37,38]; however, given that this was a retrospective study, these metrics were not available for most participants and control data and thus could not be implemented. It is also recognised that using the consensus clinical judgement with no gait analysis metrics input as a proxy gold standard is by nature subjective, and this may have introduced additional error. In addition, the choice of using the same MCID in GPS of 1.6° for the mGen, could be considered arbitrary; however, this was justified as akin to using the 5° threshold for detecting clinically relevant changes in clinical gait analysis kinematics in this and other studies [10,11,14,39]. Finally, future studies should include a larger cohort to verify the generalisability of the reported results.

## 5. Conclusions

In conclusion, the mGen model estimated both GPS and post-intervention change in GPS comparable to the PiG model. Furthermore, non-3D data based clinical judgement of outcome post-intervention aligned similarly to the longitudinal change in GPS calculated from either model. Given that the mGen lends itself to advanced musculoskeletal modelling techniques, such as muscle length modelling and estimation of muscle and joint contact forces, results from this paper constitute a first step towards the use of these techniques in the clinics, with the potential to improve the clinical decision-making process in children with cerebral palsy.

## Figures and Tables

**Table 1:** Anthropometric details for the TD cohort subgroups.

TD Groups	Age (Years)	Mass (kg)	Height (m)
Young Adults (*n* = 12)	27.8 ± 6.2	68.7 ± 14.6	1.75 ± 0.11
Teenagers (*n* = 10)	11.8 ± 1.8	44.9 ± 11.9	1.52 ± 0.14
Children (*n* = 12)	7.5 ± 1.4	28.4 ± 7.6	1.31 ± 0.12

## Data Availability

The data presented in this study are openly available in FigShare at doi:10.15131/shef.data.16982110.

## Supplementary Materials

The following are available online at https://www.mdpi.com/article/10.3390/life11121306/s1, Figure S1: Mean joint kinematic waveforms for 26 CP participants estimated with PiG and mGen models, Figure S2: Distribution of RMSD between mGen and PiG for CP cohort, Figure S3: Mean inter−trial (within session) standard deviation for kinematic waveforms from mGen and PiG for CP cohort, Figure S4: Mean inter−trial standard deviation for kinematic waveforms from mGen and PiG for TD cohort, Figure S5: Comparison of RMSD between mGen and PiG for two implementations of the mGen knee joint (3DoF and 1DoF), Table S1: Changes in GPS (ΔtGPS) estimated by mGen and PiG for CP cohort, Table S2: Right-Left Asymmetry calculated as difference between right and left limb GPS values for CP cohort, Table S3: Maximum marker tracking errors.
