# Supplementary material for: Effectiveness of Global Optimisation and Direct Kinematics in Predicting Surgical Outcome in Children with Cerebral Palsy
Source: Life (Basel). 2021 Nov 27;11(12):1306. doi: 10.3390/life11121306 (PMC8705891; doi:10.3390/life11121306)
Supplement: Supplementary file 1 [file life-11-01306-s001.zip › life-1420182-supplementary.pdf]

Supplementary Data

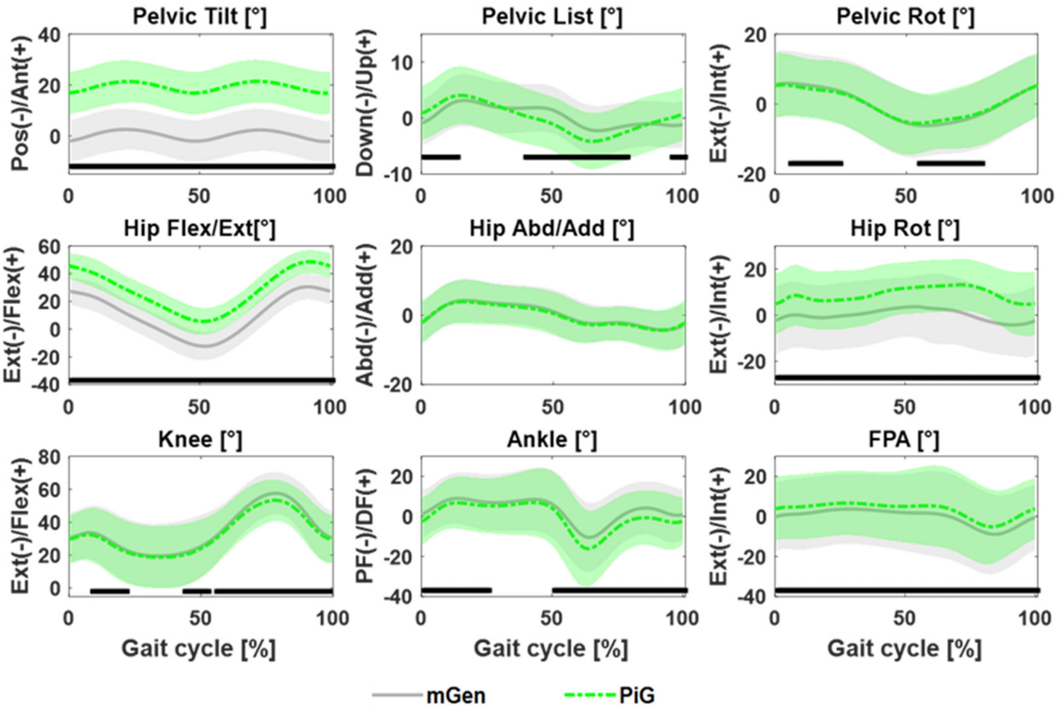

Figure S1. Mean joint kinematic waveforms for 26 CP participants estimated with PiG and mGen models. Shaded bands indicate 1SD with significant difference shown by bottom black bars.

Table S1. Changes in GPS (Δ_tGPS) estimated by mGen and PiG for CP cohort.

	RIGHT		LEFT	
**Subject**	**PiG**	**mGen**	**PiG**	**mGen**
	ΔGPS	ΔGPS	ΔGPS	ΔGPS
01	1.3	0.0	−1.6	−1.4
02	9.5	8.3	4.3	5.1
03	−3.2	−6.2	−2.6	−5.3
04	2.7	2.2	−5.4	−7.0
05	5.8	4.2	−0.7	−0.3
06	−5.3	−5.8	−8.2	−8.5
07	−6.7	−9.5	−3.7	−5.3
08	2.1	−1.2	−2.5	−5.4
09	−1.5	−0.6	−4.6	−5.4
10	−7.1	−7.6	−6.8	−1.4
11	2.2	2.1	−5.0	−3.8
12	10.3	11.0	6.0	7.5
13	0.9	−0.5	3.8	2.2
14	0.6	−1.4	−1.9	−1.3
15	2.0	1.3	3.6	2.2
16	1.1	−0.6	1.3	−0.5
17	−11.9	−8.5	−8.2	−8.9
18				
19	0.1	−2.9	−3.2	−2.1
20	−5.8	−9.5	−2.5	−5.4
21	−4.2	−4.0	−1.9	−1.5
22	−2.9	−2.0	−6.8	−5.5
23	1.2	2.0	−14.4	−12.8
24	1.5	1.8	−1.9	−2.2
25	3.4	0.4	−0.1	1.4
26	0.4	1.5	−1.2	−0.6

Table S2. Right-Left Asymmetry calculated as difference between right and left limb GPS values for CP cohort.

	**RIGHT-LEFT ASYMMETRY**			
	**Pre-FDO-SEMLS**		**Post-FDO-SEMLS**	
	PiG	mGen	PiG	mGen
	R-L Delta	R-L Delta	R-L Delta	R-L Delta
01	−8.0	−10.4	−5.2	−9.1
02	−4.7	−3.3	0.5	−0.1
03	1.0	−0.3	0.4	−1.2
04	−6.2	−7.8	1.9	1.4
05	−4.5	−4.2	2.1	0.2
06	2.0	1.5	4.9	4.2
07	5.5	7.2	2.5	3.0
08	0.0	−2.2	4.6	2.0
09	−1.9	−1.8	1.1	3.0
10	5.4	9.4	5.1	3.2
11	−6.3	−8.9	0.9	−3.0
12	−3.3	−3.3	0.9	0.2
13	3.3	3.3	0.5	0.6
14	4.5	4.8	7.0	4.7
15	2.0	2.2	0.4	1.4
16	−1.8	−2.5	−1.9	−2.5
17	5.0	2.1	1.4	2.5
18	3.0	3.3	0.0	0.0
19	−4.2	−1.9	−1.0	−2.7
20	−0.3	1.2	−3.7	−2.8
21	10.6	10.0	8.3	7.5
22	−5.7	−6.2	−1.7	−2.7
23	−11.3	−10.9	4.3	4.0
24	−2.3	1.2	1.0	5.2
25	−0.6	3.5	2.9	2.5
26	1.4	0.3	3.0	2.4

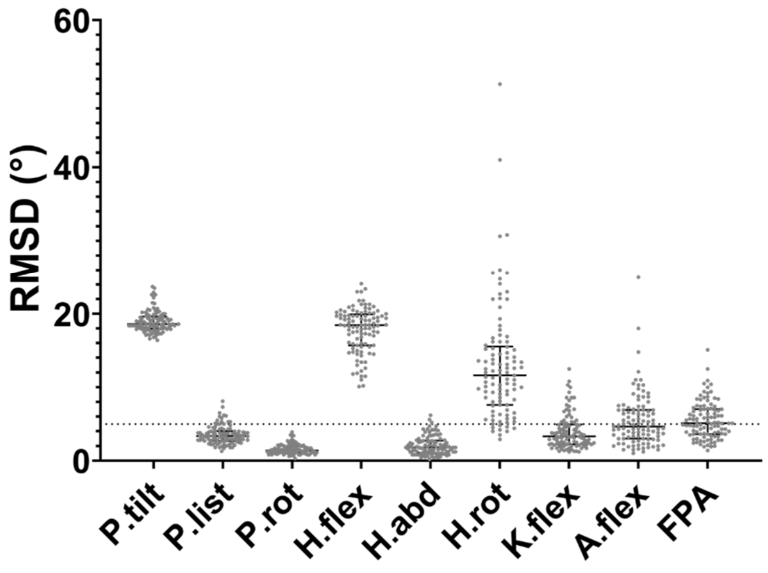

Figure S2. Distribution of RMSD between mGen and PiG for CP cohort.

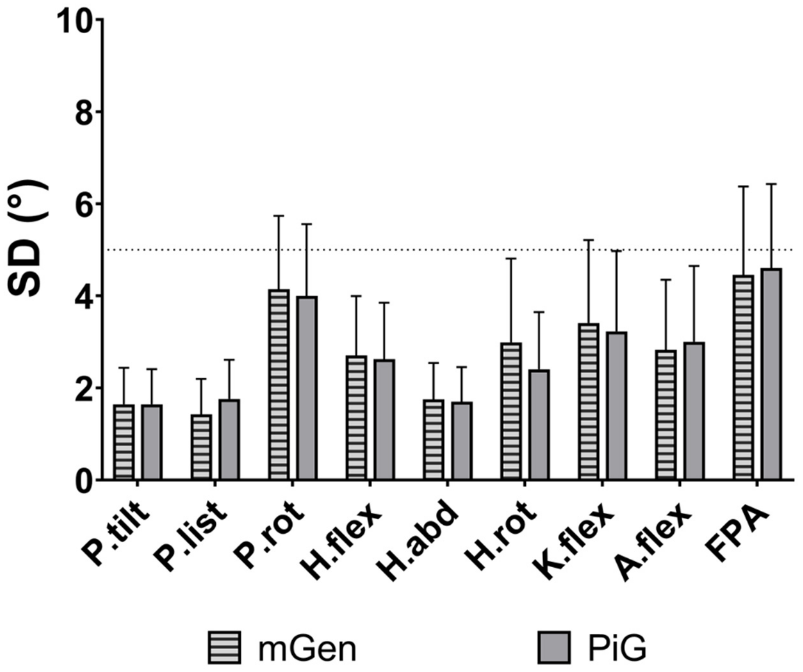

Figure S3. Mean inter−trial (within session) standard deviation for kinematic waveforms from mGen and PiG for CP cohort.

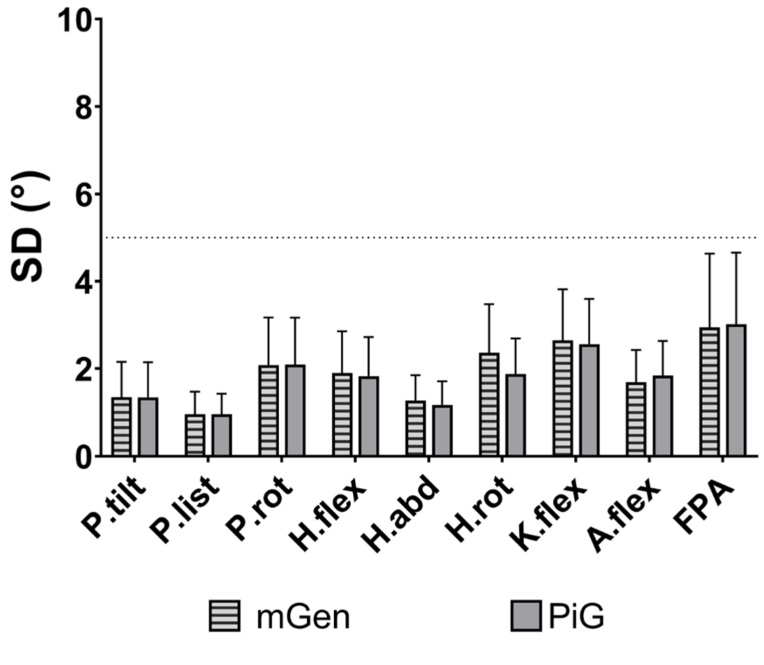

Figure S4. Mean inter−trial standard deviation for kinematic waveforms from mGen and PiG for TD cohort.

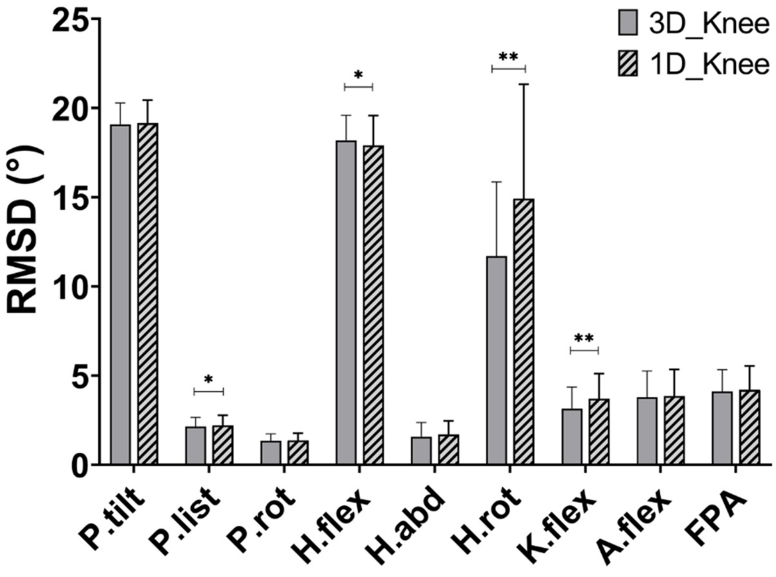

Figure S5. Comparison of RMSD between mGen and PiG for two implementations of the mGen knee joint (3DoF and 1DoF). *$p < 0.01$ **$p < 0.001$.

Table S3. Maximum marker tracking errors.

Marker	LASI	RASI	SACR	THI	KNE	TIB	ANK	HEE	TOE
Right limb	0.39 ± 0.18	0.64 ± 0.30	0.58 ± 0.25	1.88 ± 0.77	1.30 ± 0.71	1.29 ± 0.58	0.86 ± 0.35	0.96 ± 0.42	0.85 ± 0.38
Left limb	0.56 ± 0.23	0.44 ± 0.18	0.54 ± 0.24	1.74 ± 0.62	1.23 ± 0.42	1.20 ± 0.40	0.83 ± 0.30	1.04 ± 0.34	0.73 ± 0.20
